# Supplementary material for: Diversity Patterns of Epiphytic Orchids Along Elevation in the Mountains of Western Nepal
Source: Plants (Basel). 2024 Nov 20;13(22):3256. doi: 10.3390/plants13223256 (PMC11598182; doi:10.3390/plants13223256)
Supplement: Supplementary file 1 [file plants-13-03256-s001.zip › plants-3287871-supplementary.pdf]

Supplementary Table S1. List of all the epiphytic orchids with total abundance according to host species.

Epiphytic orchids	Total abundance	Hosts
Acampe rigida	28	*Sc. wallichii, Rh. arboreum, Q. semecarpifolia, Da. himalense, Ly. ovalifolia*
Agrostophyllum callosum	53	*Rh. arboreum, Q. semecarpifolia, So. cuspidata*
Bu.affine	32	*Sc. wallichii,, Rh. arboreum, Mahonia napaulensis, Rhu. wallichii*
Bu. ambrosia	15	*Sc. wallichii , Rh. arboreum, Ca. indica*
Bu. bisetum	38	*Da. himalense, Ly. ovalifolia, Vib. cylindricum, To. tiliifolia*
Bu. cylindraceum	78	*Da. himalense, Ly. ovalifolia, Al. nepalensis, Il. dipyrena, To. tiliifolia*
Bu. elatum	17	*Rh. arboreum, Q. semecarpifolia, So. cuspidata, Ly. ovalifolia , Il. dipyrena*
Bu. hirtum	19	*Sc. wallichii, Saurauia napaulensis, Rhu. wallichii*
Bu. leopardinum	118	*Rh. arboreum, Da. himalense, Ly. ovalifolia, Al. nepalensis*
Bu. muscicola	21	*Rh. arboreum, Q. semecarpifolia, Ly. ovalifolia, Q. lamellosa*
Bu. odoratissimum	78	*Sc. wallichii, Rh. arboreum, Ly. ovalifolia, Ca. indica*
Bu. polyrhizum	54	*Sc. wallichii, Rh. arboreum, Ca. indica*
Bu. reptans	105	*Sc. wallichii, Rh. arboreum, Q. semecarpifolia, Ly. ovalifolia, So. cuspidata*
Bu. retusiusculum	50	*Rh. arboreum, Q. semecarpifolia, Ly. ovalifolia, Il. dipyrena, Lin. pulcherrima*
Bu. roseopictum	44	*Da. himalense, Ly. ovalifolia, Al. nepalensis, Il. dipyrena, Vi. mullaha, Be. alnoides*
Bu. striatum	47	*Sc. wallichii, Rh. arboreum, Q. semecarpifolia, Ly. ovalifolia*
Bu. umbellatum	86	*Sc. wallichii, Rh. arboreum, Ca. indica*
Bu. viridiflorum	65	*Da. himalense, Ly. ovalifolia, Al. nepalensis, To. tiliifolia*
Bu. wallichii	108	*Da. himalense, Ly. ovalifolia, Bet. alnoides, To. tiliifolia*
Bu. yoksunense	39	*Rh. arboreum, Q. semecarpifolia, Ly. ovalifolia, Ts. dumosa*
Chiloschista usneoides	42	*Sc. wallichii, Rh. arboreum, Ca. indica, Litsea cubeba, Ca. tribuloides*
Co. corymbosa	113	*Rh. arboreum, Q. semecarpifolia , Ly. ovalifolia, Carpinus viminea*
Co. cristata	96	*Da. himalense, Ly. ovalifolia, Al. nepalensis, Rh. arboreum, Vi. mullaha*
Co. fimbriata	88	*Sc. wallichii, Ca. indica, Fi. semicordata, Sa. insigne, Ca. tribuloides*
Co. fuscescens	38	*Ly. ovalifolia, Al. nepalensis, Sc. wallichii, En. spicata, Vi. mullaha*
Co. nitida	120	*Sc. wallichii, Rh. arboreum, Ca. indica, Ca. viminea*
Co. ovalis	83	*Rh. arboreum, Q. semecarpifolia, Ly. ovalifolia, Pie. formosa*
Co. prolifera	82	*Rh. arboreum, Do. grandiflora, Q. semecarpifolia, Ly. ovalifolia, Pie. formosa, Fi. neriifolia*
Co. punctulata	91	*Rh. arboreum, Q. semecarpifolia, Dodecadenia grandiflora, Ly. ovalifolia*
Cryptochilus luteus	100	*Da. himalense, Ly. ovalifolia, Q. oxyodon, Q. lamellosa, Rh. arboreum, Be. alnoides*
Cy. × gammieanum	19	*Da. himalense, Ly. ovalifolia, Q. lamellosa, Rh. arboreum, Bet. alnoides*
Cy. aloifolium	114	*Sc. wallichii, Ca. indica, Lithocarpus elegans, Cinnamomum glanduliferum, Sapium insigne, Ca. tribuloides*
Cy. elegans	95	*Da. himalense, Persea duthiei, Q. lamellosa, Lin. pulcherrima, Bet. alnoides*
Cy. erythraeum	55	*Da. himalense, Sy. sumuntia, Q. lamellosa, Q. glauca, To. tiliifolia, Q. oxyodon, Pe. duthiei*
Cy. iridioides	92	*Rh. arboreum, Q. semecarpifolia, Ly. ovalifolia, Pie. formosa*
Cy. lancifolium	103	*Sc. wallichii, , Rh. arboreum, Ca. indica, Q. lanata, Ca. tribuloides*
Den. amoenum	139	*Sc. wallichii, Rh. arboreum, Ly. ovalifolia, Ae. indica, Sa. insigne*
Den. bicameratum	97	*Lithocarpus elegans, Rh. arboreum, Ly. ovalifolia, Taxus wallichiana*
Den. candidum	50	*Rh. arboreum, Q. glauca, Eu. cerasifolia*

Den. chryseum	70	*Sc. wallichii, Albizia chinensis, Rh. arboreum, Ly. ovalifolia, Ta. wallichiana, Q. semecarpifolia*
Den. densiflorum	144	*Sc. wallichii, Rh. arboreum, Ta. wallichiana, Ly. ovalifolia, Ae. indica*
Den. denudans	69	*Sc. wallichii, , Rh. arboreum, Ly. ovalifolia, Ae. indica*
Den. eriiflorum	65	*Sc. wallichii, Toona ciliata, Rh. arboreum, Ly. ovalifolia, Ae. indica, Al. chinensis*
Den. hookerianum	74	*Sc. wallichii, Al. chinensis, Al. nepalensis, Ca. viminea*
Den. longicornu	141	*Da. himalense, Sy. sumuntia, Ly. ovalifolia , Q. lamellosa, Eu. cerasifolia, Q. oxyodon*
Den. moniliforme	102	*Rh. arboreum, Ta. wallichiana, Myrsine semiserrata, Sa. babylonica, Sy. ramosissima, Q. semecarpifolia, Ly. ovalifolia, Q. lanata , Do. grandiflora var. grandiflora*
Den. monticola	87	*Rh. arboreum, Pr. cerasoides, Q. semecarpifolia, My. semiserrata, Ly. ovalifolia, Q. lanata*
Den. porphyrochilum	73	*Da. himalense, Ly. ovalifolia, Pe. duthiei, Eu. cerasifolia, Sy. ramosissima*
Den. transparens	39	*Sc. wallichii, Too. ciliata, Pr. venosa, Al. nepalensis, Fraxinus floribunda, Fi. semicordata*
Eria coronaria	96	*Da. himalense, Eu. cerasifolia, Ly. ovalifolia, Q. lamellosa, Rh. arboreum, Pe. duthiei*
Ga. affinis	63	*Litsea cubeba, Rh. arboreum, Q. semecarpifolia, Ts. dumosa, Do. grandiflora*
Ga. calceolaris	144	*Sc. wallichii, Rh. arboreum, Pr. venosa, Ca. indica, Rhu. javanica*
Ga. distichus	111	*Da. himalens, Ly. ovalifolia, Q. lamellosa, Lin. pulcherrima, Sy. ramosissima, Ber. aristata*
Goodyera recurva	99	*Mi. kisopa, Litsea pallens, Vi. erubescens*
Lip. bootanensis	53	*Sa. babylonica, Ju. regia, Eu. acuminata, My. semiserrata, Q. semecarpifolia, Pin. wallichiana*
Lip. resupinata	112	*Da. himalense, Ly. ovalifolia, Al. nepalensis, Rh. arboreum, Mac. pustulata*
Lip. viridiflora	75	*Sc. wallichii, Rh. arboreum, Ca. tribuloides*
Ob. acaulis	74	*Sc. wallichii, En. spicata, Sa. babylonica, Er. elliptica, Fi. semicordata, Pr. cerasoides*
Ob. caulescens	76	*Litsea cubeba, Rh. arboretum, Q. semecarpifolia , Ts. dumosa, Ly. ovalifolia ,*
Ob. falcata	106	*Da. himalense, Ly. ovalifolia, Q. lamellosa, Ae. indica, Ber. aristata*
Ob. obcordata	37	*Sc. wallichii, Too. ciliata, Fi. auriculata, Pr. cerasoides, Er. elliptica, Pyrularia edulis, Lip. cubeba*
Ot. fuscus	104	*Da. himalense, Sy. sumuntia, Sy. sumuntia*
Ot. lancilabius	118	*Er. elliptica, Da. himalense, Ly. ovalifolia, Q. lamellosa, Ae. indica,*
Panisea demissa	107	*Da. himalense Ly. ovalifolia, Q. lamellosa*
Ph. articulata	81	*Da. himalense, Ly. ovalifolia, Q. lamellosa*
Ph. imbricata	113	*Sc. wallichii, Pr. venosa, Er. elliptic, Sa. babylonica, Rh. arboreum, Ly. ovalifolia, Q. semecarpifolia*
Ph. pallida	132	*Sc. wallichii, Ca. indica, Myrica esculenta*
Pi. excavata	129	*Sc. wallichii, Deb. longifolia, Rh. arboreum, Ly. ovalifolia, Eu. acuminata, Q. semecarpifolia*
Pi. graminifolia	95	*Da. himalense, Ly. ovalifolia, Q. lamellosa, Sy. sumuntia*
Pi. spicata	143	*Sc. wallichii, Pr. cerasoides, Mi. kisopa, Ca. indica, Pin. roxburghii, Fi. semicordata*
Pl. humilis	110	*Da. himalense, Ly. ovalifolia, Ju. regia, Sy. sumuntia*
Pl. praecox	117	*Sc. wallichii, Mac. pustulata, Fi. auriculata Lour., Rh. arboreum, Ly. ovalifolia, Q. semecarpifolia*
Porpax elwesii	32	*Sc. wallichii, Deb. longifolia, Sa. insigne, Al. chinensis*
Rhynchostylis retusa	49	*Sc. wallichii, Pr. cornuta, Fi. auriculata, Ma. pustulata*

Taeniophyllum scaberulum	32	*Sc. wallichii, Ae. indica, Al. chinensis, Ju. regia, Eu. cerasifolia*
Thunia alba	59	*Sc. wallichii, Fi. religiosa, Ma. pustulata*
Va. alpina	29	*Deb. longifolia, Pr. cerasoides, Pyrus pashia, Morus serrata, Eu. acuminata*
Va. cristata	86	*Da. himalense, Eu. cerasifolia, Sy. theifolia, Ly. ovalifolia, Al. nepalensis, Rh. arboreum,*
Vandopsida undulata	89	*Da. himalense, Eu. cerasifolia, Sy. theifolia, Ju. regia, Eu. acuminata*

Abbreviations: *Ae.= Aesculus; Al.= Alnus; Ber.=* Berberis; *Bet.= Betula; Bu.= Bulbophyllum; Ca.= Castanopsis; Co.= Coelogyne; Cy.= Cymbidium; Da.= Daphniphyllum; Deb.= Debregeasia; Den.= Dendrobium; En.= Engelhardia; Er.= Eriobotrya; Eu.= Eurya; Fi.= Ficus; Ga.= Gastrochilus; Il.= Ilex; Ju= Juglans; Lin.= Lindera; Lip.= Liparis; Ly.= Lyonia; Ma.= Macaranga; Mi.= Michelia; Ob.= Oberonia; Ot.= Otochilus; Ph.= Pholidota; Pi.= Pinalia; Pie.= Pieris; Pin.= Pinus; Pl.= Pleione; Pr.= Prunus; Q.= Quercus; Rh.= Rhododendron; Rhu.= Rhus; Sa.= Salix; Sc.= Schima; So.= Sorbus; Sy.= Symplocos; To.= Toricellia; Ts.= Tsuga; Va.= Vanda; Vi.= Viburnum.*

Lit.=Litsea
